# Do Drug Accessibility and OOP Burden Affect Health-Related Quality of Life of Patients With Chronic Diseases? — EQ-5D-5L Evaluation Evidence From Five Districts in China

**DOI:** 10.3389/fpubh.2021.656104

**Published:** 2021-03-12

**Authors:** Shaoliang Tang, Ying Gong, Meixian Liu, Duoer Yang, Kean Tang

**Affiliations:** ^1^School of Health Economics and Management, Nanjing University of Chinese Medicine, Nanjing, China; ^2^Nanjing Stomatological Hospital, Nanjing Stomatological Hospital, Medical School of Nanjing University, Nanjing, China; ^3^Faculty of Science, Lund University, Lund, Sweden

**Keywords:** patients with chronic diseases, health-related quality of life, drug accessibility, OOP, CLAD model

## Abstract

**Purpose:** The dependence of patients with chronic diseases on drugs may affect their health-related quality of life (HRQoL). This study aims to assess the relationship between the direct economic burden caused by out-of-pocket (OOP) payments, drug accessibility, sociodemographic characteristics, and health-related quality of life.

**Methods:** 1,055 patients with chronic diseases from Gansu, Hebei, Sichuan, Zhejiang, and Tianjin were investigated. Data collection included basic conditions and economic and health insurance conditions of patients with chronic diseases. The CLAD and Tobit regression models were used to analyze and compare the health-related quality of life and influencing factors of patients with chronic diseases in five districts. Differentiated analysis was conducted through sub-sample regression to explore the variable health effects of patients with single and multiple diseases.

**Results:** A total of 1,055 patients with chronic diseases participated in the study, 54.4% of whom were women. The overall average utility score was 0.727, of which Sichuan Province was the highest with 0.751. Participants reported the highest proportion of pain/discomfort problems, while patients reported the least problems with self-care. The improvement of drug accessibility and the reduction of the burden of out-of-pocket expenses have significant positive effects on HRQoL. Various sociodemographic factors such as age and gender also have significant impact on HRQoL of patients with chronic diseases. HRQoL of patients with multiple chronic diseases is more affected by various influencing factors than that of patients with single disease.

**Conclusion:** In order to improve the quality of life of patients with chronic diseases, it is of great importance to ensure the accessibility of drugs and reduce patients' medication burden. Future focus should shift from preventing and controlling chronic diseases as individual diseases to meeting the comprehensive health needs of people suffering from multiple diseases.

## Introduction

Chronic diseases are also called non-communicable diseases (NCD). From a macro perspective, this type of disease causes 41 million deaths each year, equivalent to 71% of deaths worldwide. 15 million people die between the ages of 30 and 69, and such premature deaths mostly occur in low- and middle-income countries (1). These countries also suffer from direct losses in productivity and medical care due to the high incidence of chronic diseases, which in turn brings a huge economic burden to the country and inhibits its sustainable development (2). Personally, patients with chronic diseases suffer from high drug costs and lost productivity, and the quality of life of patients is significantly affected (3). Chronic diseases have brought huge direct economic burden to patients and their families (4).

Health is a solid foundation for all-round human development and social progress, and a necessary condition for promoting economic and social construction. At present, there are a large number of people suffering from chronic diseases in China, and the incidence and mortality of chronic diseases remain high. Chronic diseases are endangering people's health. In October 2016, ≪the Plan of Health China 2030≫ proposed: “It is necessary to strengthen the intervention of health problems and their influencing factors at different stages of life, and comprehensively safeguard people's health” (5). With the change of disease spectrum and medical models, traditional indicators for evaluating the relationship between health and disease, such as morbidity, cure rate, mortality, etc., can no longer fully and deeply reflect the health status of patients with chronic diseases. It is necessary to evaluate the health-related quality of life of patients with chronic diseases from multiple perspectives such as physical, mental, and social environment. HRQoL is a multidimensional concept involving general functional status and symptoms related to disease or treatment (6). It involves the functional stability of a person's mental, physical, and social dimensions, and can fully reflect the interaction between the individual and the society (7). HRQoL assessment uses various mental and physical indicators to measure a person's perceived quality of life, and is increasingly considered to be an important result of chronic diseases (8). Therefore, this study measured the HRQoL of patients with chronic diseases to enable us to understand the health characteristics of this population.

For the HRQoL of patients with chronic diseases, we choose the perspective of drug accessibility and OOP burden for research. Compared with patients with non-chronic diseases, the health quality monitoring and long-term medication of patients with chronic diseases require financial support (9). These health-related costs will cause cost-related medication non-adherence (CRN), and records show that patients will delay dispensing or even reduce or skip medication, which easily put patients at risk of complications (10), such as diabetes and cancer (11). The economic burden of medical expenditure will cause unpredictable consequences for patients. The Financial Toxicity caused by high and sustained medical expenses will have significant impact on the psychology and income of patients with chronic diseases (12), and these effects will be reflected in the HRQoL of patients. In addition, the Canadian federal government also advocates measuring patients' accessibility and affordability of prescription drugs (13). Therefore, this study also focuses on the impact of drug accessibility and affordability of out-of-pocket expenses on HRQoL in terms of medication and treatment characteristics of patients with chronic diseases.

The EQ-5D-5L scale consists of five questions/domains, which are used to detect health status (14). The Chinese version of the scale has been found to have high applicability. It is reliable, effective and appropriate to the cultural and psychological characteristics of Chinese people (15, 16). Few studies have attempted to explore this scale in the Chinese population with chronic diseases, and this study can make marginal contribution. In addition, most scholars currently focus on the research on HRQoL of patients with single disease such as hypertension and cancer. Few studies focus on chronic disease groups and discuss the drug accessibility and the burden of OOP as research perspectives. Therefore, this study aims to analyze the quality of life and its influencing factors of patients with chronic diseases in five districts (Gansu, Hebei, Sichuan, Zhejiang, and Tianjin) of China by using EQ-5D-5L and its newly developed Chinese value set. On the basis of analyzing the status of health-related quality of life and health differences, the Clad model is used to analyze whether drug accessibility and OOP burden will affect the health of patients with chronic diseases in five districts. On the basis of categorizing patients' disease status into single disease and multiple diseases, the health improvement effects of each influencing factor were investigated separately, and practical and feasible suggestions were put forward for improving the health-related quality of life of patients with chronic diseases in China.

## Methods

### Study Design and Patients

The study design, data collection, and interpretation were supported by several funding projects. Due to the imbalance of regional development in China, this study adopted a combination of stratified sampling and random sampling to ensure the consistency of subjects. First, we determined the provinces to be investigated based on geographical location, the GDP per capita level and health resources allocation. Second, we selected one tertiary hospital and two secondary hospitals from each province. Third, we selected a number of patients with chronic diseases in each hospital as the survey subjects. The diseases of patients with chronic diseases included in this study were mainly chronic non-communicable diseases such as hypertension, cardiovascular and cerebrovascular diseases, and cancer. At the same time, some samples were patients with diabetes, chronic bronchitis, asthma, chronic pharyngitis, gastritis, and other diseases.

In 2018, among 31 provinces and cities in mainland China, Zhejiang's GDP per capita was 101,813 RMB, ranking fourth. Tianjin's GDP per capita was 85,757 RMB, ranking seventh. Hebei's GDP per capita was 43,108 RMB, ranking 25th. We chose Zhejiang Province, Tianjin City, and Hebei Province as the sample areas with relatively high and low economic levels in the eastern region, which could better represent the eastern region of China. In the western region, Sichuan Province and Gansu Province ranked 19th (51556 RMB) and 31st (30797 RMB) in GDP per capita, respectively, and the economic gap was obvious. Both provinces had implemented health poverty alleviation policies, such as Inclined Medical Insurance and the Social Security System (17, 18), and could better represent the western region of China. In addition, in terms of health resources allocation, the growth rate of the number of public health personnel per thousand population in China showed a state of polarization in 2018. Zhejiang and Sichuan showed a positive growth, while Tianjin, Hebei, and Gansu showed a negative growth, which could represent the overall status quo of health resource allocation in China. The research team selected five representative provinces and cities (Gansu, Hebei, Sichuan, Zhejiang, and Tianjin) as the sample provinces in eastern and western regions. The selected sample hospitals in Gansu Province were Gansu Provincial Hospital of Traditional Chinese Medicine and Longxi Hospital of TCM. In Hebei Province, the selected sample hospitals were Hebei General Hospital and Affiliated Hospital of Chengde Medical University. The selected sample hospitals in Sichuan Province were Sichuan Provincial People's Hospital (Eastern Hospital), Chengdu Eastern District Hospital, Chengdu Modern Hospital and Kangfu Kidney Hospital of Chengdu. In Zhejiang Province, the selected sample hospitals were the Affiliated Hospital of Hangzhou Normal University, two community hospitals in Yuhang District of Hangzhou, and Ningbo Yinzhou People's Hospital. The selected sample hospitals in Tianjin were First Teaching Hospital of Tianjin University of Traditional Chinese Medicine, Tianjin Cancer Hospital, Second Affiliated Hospital of Tianjin University of TCM, and People's Hospital of Nankai University. Patients with chronic diseases in the above-mentioned hospitals were randomly selected as the investigation objects, and the questionnaire survey in the form of interview was carried out.

1,440 questionnaires were distributed, and 1,356 were recovered. The recovered questionnaires were cleaned according to the following inclusion and exclusion criteria, and finally 1,055 valid questionnaires were obtained, among which there were 325 chronic disease patients in Gansu, 202 chronic disease patients in Hebei, 193 chronic disease patients in Sichuan, 211 chronic disease patients in Zhejiang, and 124 chronic disease patients in Tianjin.

The inclusion criteria were: the respondent had at least one chronic disease diagnosed by the doctor, and had the willingness and ability to understand the questionnaire.

The exclusion criteria were: the respondent was too weak to communicate in words, or had certain mental barriers.

We used a simple random sampling formula to calculate the sample size of patients with chronic diseases in five places because more parameters were needed in the stratified sampling calculation formula (19). After obtaining the required total sample size, we used probability proportional scale sampling (PPS) to allocate the sample size of the selected hospital.

In simple random sampling, the margin of error and the standard deviation of the estimator are usually used to determine the required sample size (20):

$$\begin{array}{l} n=\frac{t^{2}\sigma^{2}}{{\Delta_{\bar{x}}}^{2}+\frac{t^{2}\sigma^{2}}{N}} \end{array}$$

($\Delta_{\bar{x}}$ is the allowable error limit, t is the statistic of a given confidence level, *N* is the population size, and σ^2^ is the population variance estimate.)

Generally, the variance p(1-p) can be substituted into the formula. Take *p* = 0.5 to maximize the variance of the population. Without considering the finite population correction factor, $n_{1}=\frac{t^{2}p(1-p)}{\Delta^{2}}=\frac{{\left(1.96\right)}^{2}(0.5)(1-0.5)}{{\left(0.05\right)}^{2}}=384$. After adjusting the population size, $n_{2}=n_{1}\frac{N}{N+n_{1}}\approx 384$. Therefore, without considering the response rate, the sample size required for the survey is 384. Obviously, the sample size of this study met the requirements.

### Variable Description

Quality control measures were implemented during the data collection process. The data was collected by well-trained team members through structured standard questionnaires. In order to ensure the accuracy and completeness of the collected data, each hospital had assigned 2 quality controllers to supervise the interviewers on site, and regularly checked 100% of the questionnaires and 50% of the records after the interview.

Many scholars such as Addis and Cortesi have studied HRQoL through demographic characteristics variables (21, 22). In 1997, the life course framework proposed by Kuh and Schlomo explained that health was a dynamic process, which was affected by the combined effect of biological and social factors, which inspired us to understand a wider range of determinants of health, including the impact of health resource exposure and economic pressure (23). At the same time, previous studies had proved that implementing appropriate interventions to deal with more inclusive social determinants of health problems (access to health services) was under increasing pressure (24). For patients with chronic diseases, the direct economic burden caused by out-of-pocket (OOP) payments had negative impact on HRQoL (25). Out-of-pocket costs for drugs accounted for a large part of out-of-pocket costs, which was catastrophic (26). And from a fair point of view, the national drug system providing patients with professional drugs or high-cost drugs should take the individual's situation into account. It was necessary to consider the accessibility of professional drugs and the affordability of high-cost drugs (27). Based on the above, the research independent variables include conventional social demographic characteristics and the level of drug accessibility and the affordability of out-of-pocket (OOP) expenses for patients with chronic diseases.

As shown in Table 1 Social demographic characteristics include: age, gender, BMI category (wasting BMI < 18.5, normal 18.5 ≤ BMI < 24, overweight BMI ≥ 24) (28), marital status (married, unmarried/divorced/widowed), education level (Below junior high school education, junior high school education, and above), income level (0–999, 1,000–1,999, 2,000–2,999, 3,000, and above), medical insurance category (Urban Resident Basic Health Insurance, Urban Workers Basic Health Insurance, New Rural Cooperative Health Insurance, Others), health files (yes, no, don't know). The level of drug accessibility is measured by the question “Can the required drugs be purchased in public health institutions?”, and the affordability level of out-of-pocket expenses for patients with chronic diseases is measured by the question “Do you think the burden of drug costs is heavy?”.

**Table 1:** Variable description.

**Variable**	**Description of variable setting**	**Mean**	**Standard deviation**
Age	0–49 = 1, 50–59 = 2, 60–69 = 3, 70, or more = 4	2.75	1.124
Gender	Male = 1, Female = 2	1.54	0.498
BMI	Underweight (BMI < 18.5) = 1, Normal (18.5 ≤ BMI < 24) = 2, Overweight (BMI ≥ 24) = 3	1.21	0.668
Marital status	Unmarried/Divorced/Widowed = 1, Married = 2	1.82	0.384
Education level	Below junior high school education = 1, Junior high school, and above = 2	1.30	0.460
Income level	0–999 = 1, 1000–1999 = 2, 2000–2999 = 3, 3000, and more = 4	2.46	1.221
Health insurance	Urban residents basic health insurance = 1, Urban workers basic health insurance = 2, New rural cooperative health insurance = 3, Others = 4	2.11	0.978
Health file	Yes = 1, No = 2, Unknown = 3	2.02	0.707
Drug accessibility	Can medicines needed be purchased in public medical and health institutions? Very inconsistent 1-2-3-4-5 Very consistent	4.33	0.902
OOP level	Do you think the cost of medicine is heavy? Very heavy 1-2-3-4-5 Very light	2.66	1.196
Chronic disease	Monopathy = 1, Multimorbidity = 2	1.17	0.378
Province	Gansu = 1, Hebei = 2, Sichuan = 3, Tianjin = 4, Zhejiang = 5	2.71	1.503
Mobility	Extremely difficulty 1-2-3-4-5 No difficulty	0.63	0.483
Self care	Extremely difficulty 1-2-3-4-5 No difficulty	0.66	0.475
Usual activities	Extremely difficulty 1-2-3-4-5 No difficulty	0.62	0.485
Pain or discomfort	Extremely difficulty 1-2-3-4-5 No difficulty	0.32	0.467
Anxiety or depression	Extremely difficulty 1-2-3-4-5 No difficulty	0.41	0.493
Utility	Calculated based on EQ-5D-5L and its newly developed Chinese value set, the maximum value is 1, the minimum value is −0.391.	0.727	0.345

### EQ-5D Utility Value Measurement

EQ-5D-5L Scale (European Five-Dimensional Health Scale): Containing five dimensions and five levels under each dimension. The five dimensions are Mobility (MO), Self-care (SC), Usual Activities (UA), Pain/Discomfort (PD), and Anxiety/Depression (AD); The five levels in each dimension are arranged in order of difficulty. In this study, the health utility value is used to evaluate the health-related quality of life of patients with chronic diseases in five districts of Gansu, Hebei, Sichuan, Zhejiang, and Tianjin. The health utility value of this study uses the Chinese version of the EQ-5D-5L value set (29, 30) to transform the values of the five dimensions of MO, SC, UA, PD, and AD (Table 2).

**Table 2:** Chinese version of EQ-5D-5L value set.

	**MO**	**SC**	**UA**	**PD**	**AD**
No difficulty	0	0	0	0	0
Mild difficulty	0.066	0.048	0.045	0.058	0.049
Moderate difficulty	0.158	0.116	0.107	0.138	0.118
Severe difficulty	0.287	0.210	0.194	0.252	0.215
Extremely difficult	0.345	0.253	0.233	0.302	0.258

### Statistical Analysis

In this study, the health utility value of patients with chronic diseases is between −0.391 and 1, showing a skewed distribution. The error term does not meet the requirement of normality, which is a typical limited dependent variable situation. The Tobit model is considered to be one of the effective methods for processing such data. At the same time, the Tobit model has better effect than the OLS regression in solving the ceiling effect of the EQ-5D scale. However, the Tobit model requires the random error term to satisfy normality and homoscedasticity, otherwise the final regression result will be biased.

Therefore, the semi-parametric research method in the Tobit model is chosen to be used in this research. The semi-parametric method has lower dependence on the specific distribution of the model, better robustness, and less demand for sample size (31, 32), which can avoid interference from random error terms and obtain more reliable regression result. The Censored Least Absolute Deviation Model (CLAD) proposed by James Powell in 1984 was a semi-parametric estimation method of the Tobit model. Through systematic literature research, Dakin, H found that when using the EQ-5D scale to study the health utility value, the usage rate of the CLAD model was close to 20% (33). The CLAD model has been applied to the treatment evaluation of cancer patients and the HRQoL evaluation of asthma patients (34, 35).

The CLAD model can be expressed as:

$$y_{i}=\text{max}(0,x_{i}^{\prime }\beta +\epsilon_{i})$$

The objective function of CLAD is the sum of absolute deviations: ${\text{min}}_{\beta }\sum_{i=1}^{n}\left|y_{i}-max(0,x_{i}^{\prime }\beta +\epsilon_{i})\;\right|$.

This study uses Excel16.16.5 to input survey data, and Stata15.1 for descriptive analysis and regression analysis. The measurement data in the statistical description part of the study is represented by mean, median, and quartile. The overall situation of the sample is represented by the number of cases and percentage. Based on the five dimensions of mobility, self-care, usual activities, pain or discomfort, anxiety or depression, patients with chronic diseases are divided into healthy groups and problem groups and data distribution is visualized. Compare the health-related quality of life of patients with chronic diseases in Gansu, Hebei, Sichuan, Zhejiang, and Tianjin. In the analysis of influencing factors, the CLAD model is used for regression analysis based on the overall dimension (health utility value). On the basis of dividing chronic disease patients into single disease patients and multiple diseases patients, the health improvement effects of each influencing factor are investigated, respectively.

## Results

### Patient Characteristics of Five Districts

As shown in Appendix 1, patients with chronic diseases over 70 years old account for about one-third of the sample population. In terms of gender, the proportion of women suffering from chronic diseases is 7.8% higher than that of men. According to the BMI index, there are 536 (50.8%) patients with chronic diseases of normal size in the five districts. In the same level comparison, the proportion of chronic emaciation patients is the highest in Tianjin, accounting for 30.6%; Overweight patients with chronic diseases are most distributed in Hebei (44.1%). In terms of marital status, the majority of chronic patients in five districts are married, accounting for 82.1% of the total; in the case of non-married, chronic patients in Tianjin account for 48.4% of the total. In terms of education level, the majority of chronic disease patients with junior high school education and below account for 69.7%; among chronic patients with higher degree, Hebei sample has the highest proportion of 40.3%. In the aspect of average monthly income, the sample distribution of each income segment is relatively balanced, among which the proportion of patients in the high-income range (3000 RMB and above) in Zhejiang Province is relatively high, reaching 48.8%. Among all the samples of patients with chronic diseases, 94.1% have purchased urban residents' health insurance, urban workers health insurance or new rural cooperative health insurance. In the variable of whether to have a health record, only 23.9% of patients with chronic diseases have their own health records.

### Five-Dimensional Distribution of Patient Characteristics

**Figure 1:**
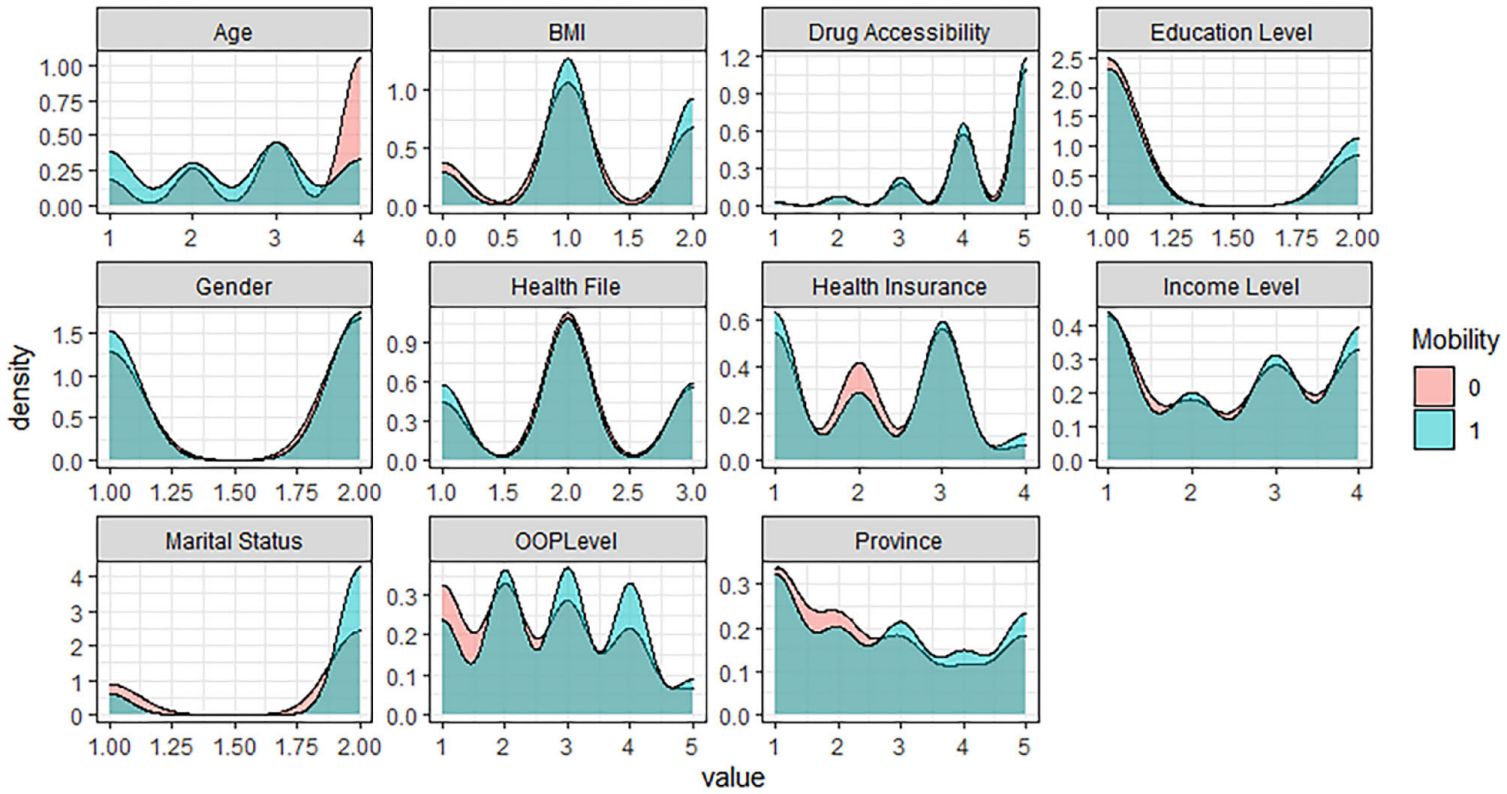
Distribution of different characteristics in the dimension of Mobility.

**Figure 2:**
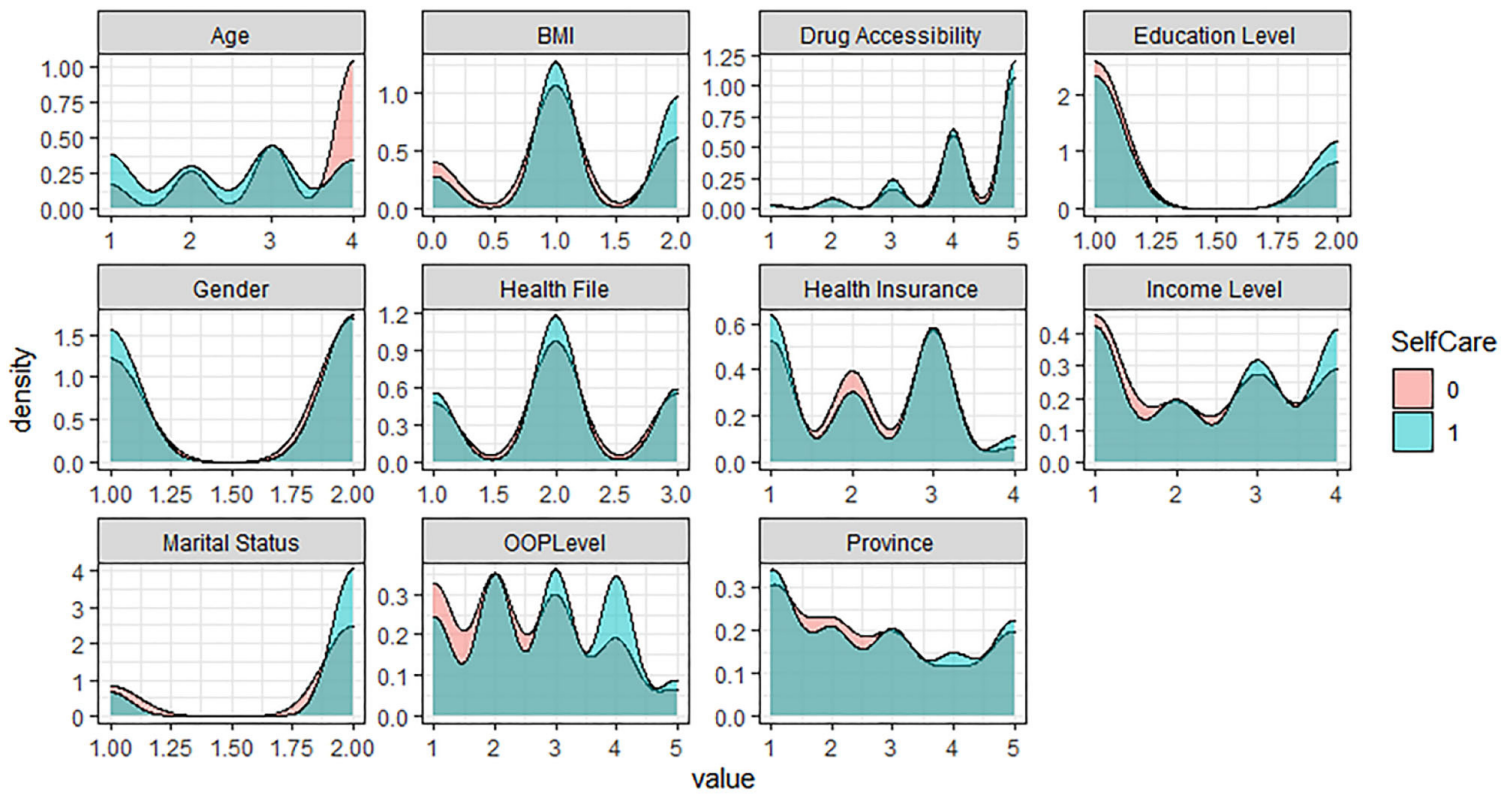
Distribution of different characteristics in the dimension of Selfcare.

**Figure 3:**
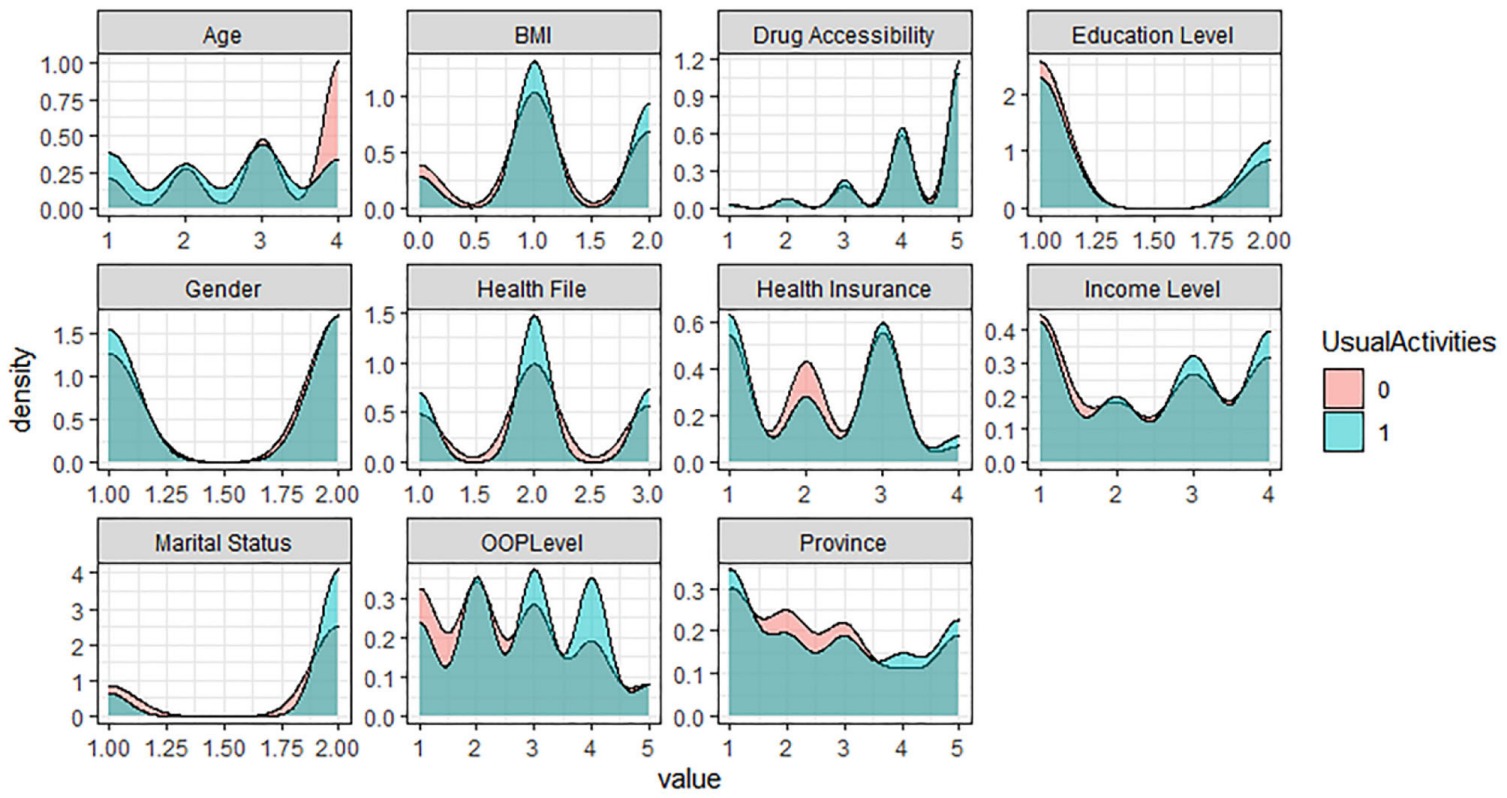
Distribution of different characteristics in the dimension of Usual Activities.

**Figure 4:**
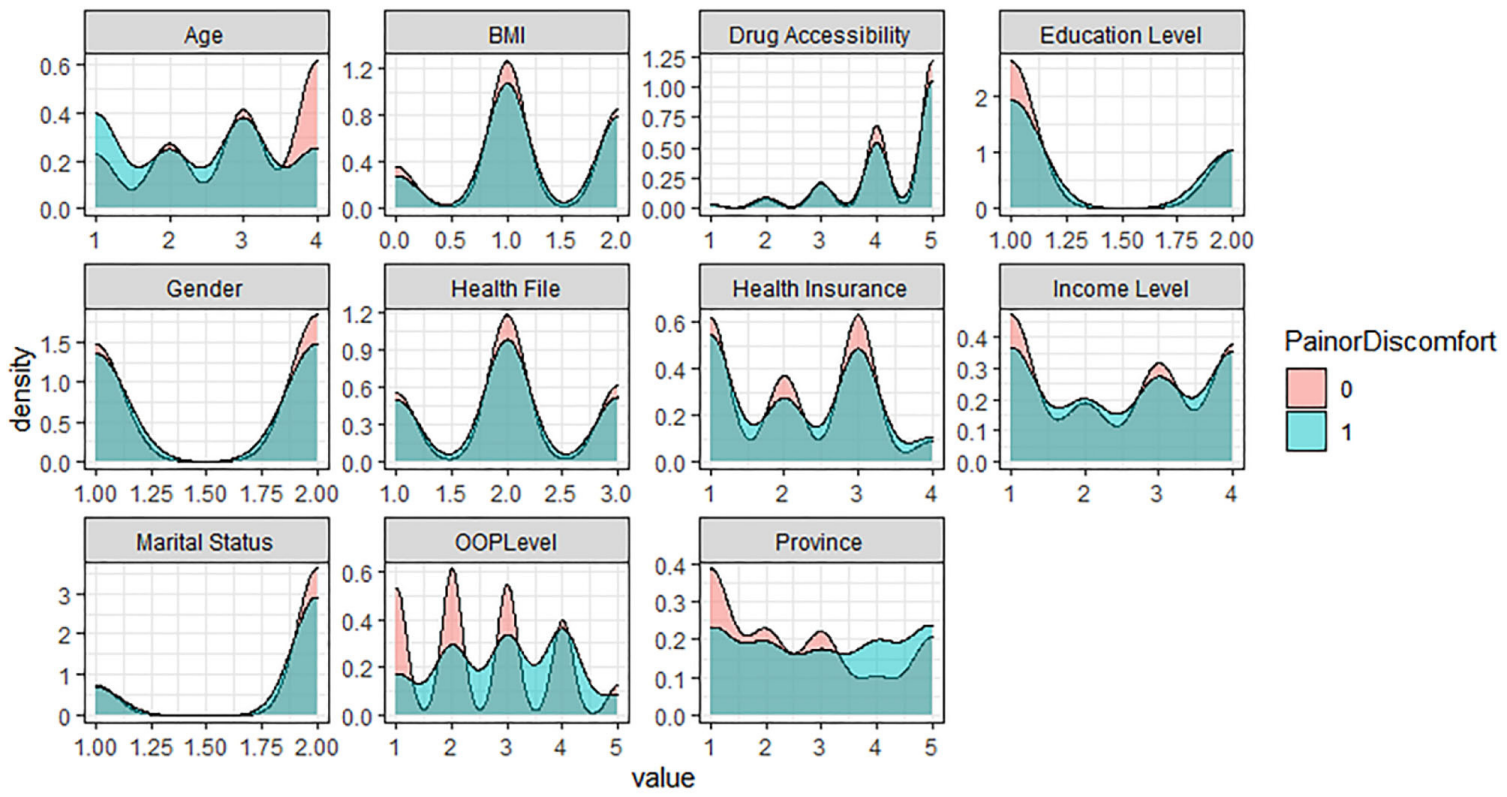
Distribution of different characteristics in the dimension of Pain (Discomfort).

**Figure 5:**
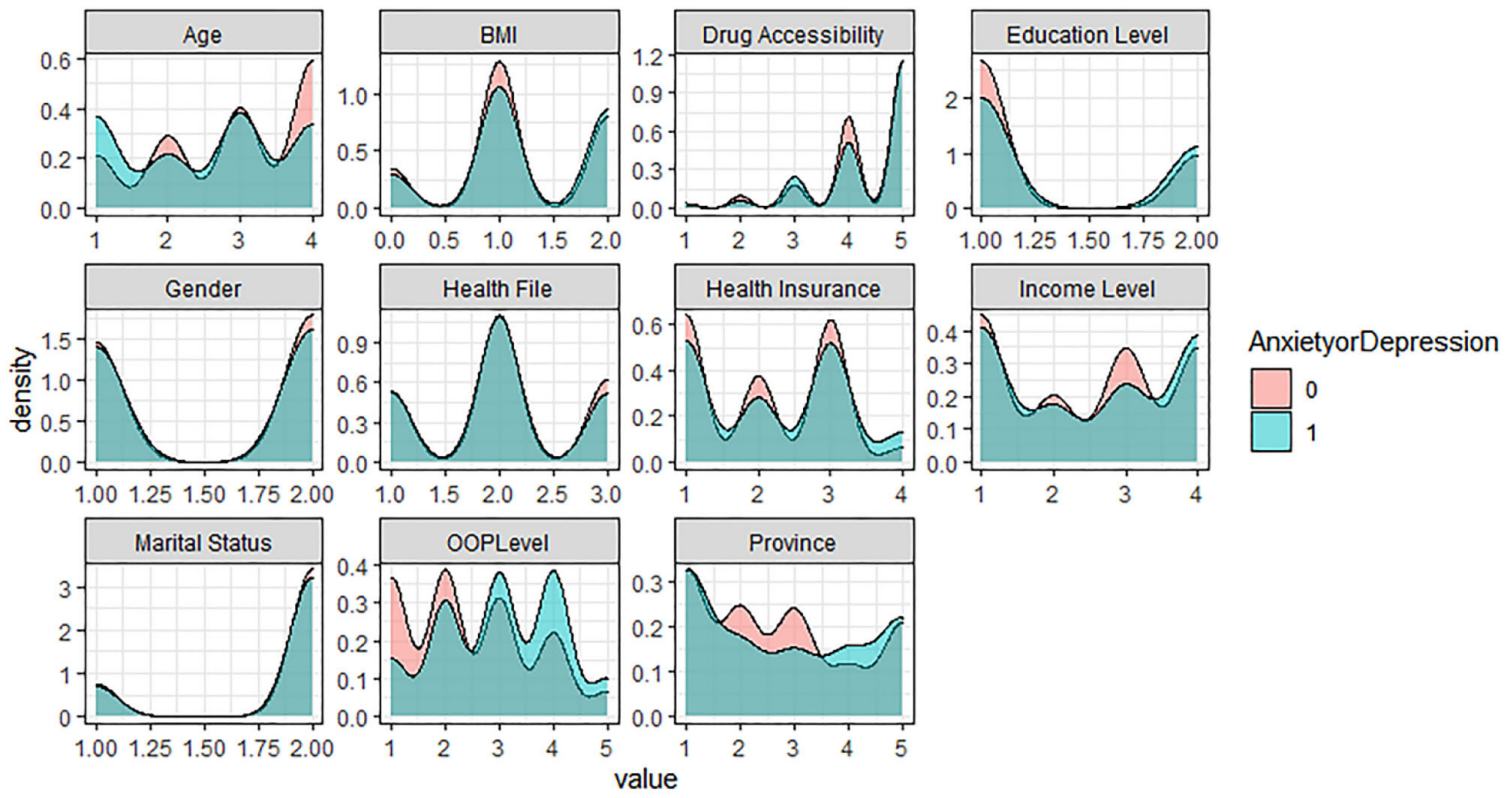
Distribution of different characteristics in the dimension of Anxiety (Depression).

In the five levels of the EQ-5D Scale, no difficulty is assigned as 1, representing the healthy group; the other four levels are assigned as 0, representing the problem group. Based on R software, the density curve graph was used to visualize the data distribution of each variable in the healthy group and the problem group in five different dimensions. As shown in Figures 1–5 below, Age and OOP Level have obvious differences in five different dimensions. Health Insurance and province have major differences in UA, PD, and AD dimensions. Marital Status has differences in the dimensions of MO, SC, UA, and PD. Health File has certain differences in the UA and PD dimensions. The differences in the data distribution of other characteristics in the five dimensions are not very obvious.

Table 3 shows that the highest health utility value for patients with chronic diseases is 1, and the lowest is −0.391. The lowest health utility value of patients with chronic diseases in Hebei Province is −0.251, and the value of the others is −0.391. From an average point of view, patients with chronic diseases in Gansu and Zhejiang have poor quality of life, while patients with chronic diseases in Sichuan and Tianjin have better health-related quality of life.

**Table 3:** Comparison of HRQOL in PCD in five districts.

		**Min value**	**Max value**	**Mean**	**Standard deviation**	**Quartile**
Health utility	Gansu	−0.391	1	0.72143	0.33148	(0.654; 0.848; 0.942)
value	Hebei	−0.251	1	0.72530	0.31466	(0.585; 0.831; 0.951)
	Sichuan	−0.391	1	0.75089	0.31700	(0.719; 0.848; 0.951)
	Zhejiang	−0.391	1	0.71799	0.39335	(0.634; 0.893; 1)
	Tianjin	−0.391	1	0.72654	0.38608	(0.640; 0.893; 1)
	Total	−0.391	1	0.72747	0.34470	(0.654; 0.876; 0.951)

### Factors Affecting Health-Related Quality of Life: An Assessment of Drug Accessibility and OOP Burden

The health utility value of patients with chronic diseases in five districts is used as the dependent variable. All groups in the basic personal situation of patients, the level of drug accessibility and the affordability level of out-of-pocket expenses are used as independent variables to establish a regression model.

The CLAD regression results are shown in Table 4. The higher the level of drug accessibility, the higher HRQoL utility value of patients with chronic diseases is (*p* < 0.001); The affordability of out-of-pocket expenses for chronic disease patients is also positively correlated with HRQoL utility value (*p* < 0.001). In terms of socio-demographic characteristics, age (*p* < 0.001), gender (*p* < 0.01), marital status (*p* < 0.001), BMI, education level (*p* < 0.001), income (*p* < 0.001), health files (*p* < 0.001), and provinces are significantly related to the HRQoL utility score of chronic disease patients.

**Table 4:** Influencing factors of HRQOL in PCD in five provinces and cites based on CLAD model.

**Variable**	**Coef**.	**Std. Err**	***t* Value**	***P* Value**	**95% Conf. Interval**	
**Drug accessibility**	0.00638	0.0078039	0.00243	<0.01	−0.010514	0.0204552 (N)
					−0.0094037	0.0201968 (P)
					−0.0136449	0.0188151 (BC)
**OOP level**	0.0387	0.0056144	0.00189	<0.001	0.0173811	0.0396613 (N)
					0.0198693	0.0392077 (P)
					0.0189541	0.0386785 (BC)
**Gender** (based on Male)						
Female	−0.0137	0.0135819	0.00435	<0.01	−0.0302315	0.0236672 (N)
					−0.0290861	0.0268571 (P)
					−0.0290861	0.0268571 (BC)
**Age**	−0.00482	0.0005752	0.000132	<0.001	−0.0051662	−0.0028837 (N)
					−0.0054801	−0.0031183 (P)
					−0.0046802	−0.0025155 (BC)
**BMI** (based on underweight)						
Normal	0.0406	0.0333965	0.00653	<0.001	0.0162456	0.1487775 (N)
					0.0224149	0.1406623 (P)
					0.0347615	0.2167522 (BC)
Overweight	0.0744	0.035125	0.00693	<0.001	0.0104422	0.1498333 (N)
					0.0147658	0.1442673 (P)
					0.0219815	0.1970143 (BC)
**Marital status** (based on unmarried/ divorced/widowed)						
Married	0.113	0.0348526	0.00616	<0.001	0.0208828	0.1591932 (N)
					0.0463194	0.1782493 (P)
					0.0336279	0.1545755 (BC)
**Education level** (based on below junior high school education)						
Junior high school	−0.0204	0.0208588	0.00503	<0.001	−0.047949	0.0348276 (N)
and above					−0.0514564	0.0246498 (P)
					−0.0446476	0.0386269 (BC)
**Income level** (based on 0–999)						
1000–1999	0.0351	0.0217253	0.00714	<0.001	−0.0431101	0.0431053 (N)
					−0.02897	0.0518653 (P)
					−0.0305684	0.0461037 (BC)
2000–2999	0.0465	0205374	0.00688	<0.001	−0.0369386	0.0445625 (N)
					−0.039424	0.0440169 (P)
					−0.0413443	0.0415212 (BC)
3000 and more	0.0465	0.022541	0.00747	<0.001	−0.0499566	0.0394959 (N)
					−0.0515417	0.0407991 (P)
					−0.0519541	0.0295373 (BC)
**Health insurance** (based on Urban residents basic health insurance)						
Urban workers basic health insurance	0.0257	0.0177376	0.00679	<0.001	−0.0216205	0.0487701 (N)
health insurance					−0.0106501	0.0583563 (P)
					−0.0131266	0.044338 (BC)
New rural cooperative	0.00569	0.0223578	0.00684	>0.05	−0.0343377	0.0543876 (N)
health insurance					−0.0317946	0.060512 (P)
					−0.0289373	0.0655845 (BC)
Others	−0.0548	0.0261476	0.00990	<0.001	−0.0234768	0.0802884 (N)
					−0.0302726	0.0697042 (P)
					−0.0176244	0.0875789 (BC)
**Health file** (based on Yes)						
No	0.0111	0.0179018	0.00569	>0.05	−0.0347315	0.0363106(N)
					−0.0403512	0.0384335 (P)
					−0.0436282	0.0290365 (BC)
Unknown	−0.0240	0.019361	0.00630	<0.001	−0.0793121	−0.0024794 (N)
					−0.0837331	−0.0003717 (P)
					−0.0846352	−0.0056006 (BC)
**Province** (based on Gansu province)						
Hebei	0.0417	0.016841	0.00599	<0.001	−0.0157497	0.0510826 (N)
					−0.0164999	0.050216 (P)
					−0.0036802	0.0558435 (BC)
Sichuan	0.0204	0.0198693	0.00674	<0.01	−0.0147695	0.0640806 (N)
					−0.0101905	0.0702229 (P)
					−0.0205779	0.0504828 (BC)
Tianjin	0.0554	0.0229514	0.00812	<0.001	0.0019729	0.0930539 (N)
					0.0087397	0.0911247 (P)
					0.0086899	0.0879101 (BC)
Zhejiang	0.0860	0.0186203	0.00664	<0.001	0.0226057	0.0964991 (N)
					0.030044	0.1016417 (P)
					0.0186889	0.0864179 (BC)
_cons	0.763	0.0557844	0.0191	<0.001	0.760537	0.9819137 (N)
					0.7483205	0.9789477 (P)
					0.7760213	0.9971853 (BC)

*N, normal; P, percentile; BC, bias-corrected*.

This shows that, in addition to sociodemographic variables, the accessibility and affordability of medicines are crucial in determining the quality of life of patients with chronic diseases.

Wooldridge (2010) pointed out that if the Tobit model was correctly set, the estimated results of CLAD and Tobit should be similar. From this perspective, the estimation result of CLAD can be roughly regarded as a test of the Tobit model. Table 5 shows that the CLAD estimated value is quite different from the Tobit estimated value. The two estimation methods have large differences in the significance of the variables. It is considered that the CLAD model should be used instead of the Tobit model because it is not reasonable enough.

**Table 5:** Influencing factors of HRQOL in PCD in five districts: comparison of CLAD model and Tobit model results.

**Variable**	**Tobit**	**CLAD**
**Drug accessibility**	0.0215^* ^ (0.0109)	0.00638^**^ (0.00243)
**OOP level**	0.0412^***^ (0.00828)	0.0387^***^ (0.00189)
**Gender** (based on Male)		
Female	−0.00661 (0.0195)	−0.0137^**^ (0.00435)
**Age**	−0.00704^***^ (0.000581)	−0.00482^***^ (0.000132)
**BMI** (based on Underweight)		
Normal	0.145^***^ (0.0304)	0.0406^***^ (0.00653)
Overweight	0.163^***^ (0.0318)	0.0744^***^ (0.00693)
**Marital status** (based on unmarried/divorced/widowed)		
Married	0.137^***^ (0.0277)	0.113^***^ (0.00616)
**Education level** (based on Below junior high school education)		
Junior high school and above	0.00632 (0.0228)	−0.0204^***^ (0.00503)
**Income level** (based on 0–999)		
1000–1999	0.0520 (0.0318)	0.0351^***^ (0.00714)
2000–2999	0.00660 (0.0306)	0.0465^***^ (0.00688)
3000 and more	0.0224 (0.0331)	0.0465^***^ (0.00747)
**Health insurance** (based on Urban residents basic health insurance)		
Urban workers basic health insurance	0.0418 (0.0280)	0.0257^***^ (0.00679)
New rural cooperative health insurance	0.0245 (0.0295)	0.00569 (0.00684)
Others	0.0537 (0.0475)	−0.0548^***^ (0.00990)
**Health file** (based on Yes)		
No	0.00565 (0.0257)	0.0111 (0.00569)
Unknown	−0.0525 (0.0281)	−0.0240^***^ (0.00630)
**Province** (based on Gansu province)		
Hebei	0.00787 (0.0284)	0.0417^***^ (0.00599)
Sichuan	0.0489 (0.0299)	0.0204^**^ (0.00674)
Tianjin	0.00171 (0.0368)	0.0554^***^ (0.00812)
Zhejiang	0.0368 (0.0297)	0.0860^***^ (0.00664)
_cons	0.667^***^ (0.0855)	0.763^***^ (0.0191)

t statistics in parentheses

* *p < 0.05*,

** *p < 0.01*,

*** *p < 0.001*.

### Differential Health Effects of Monopathy and Multimorbidity

In China, people suffering from multimorbidity are inseparable from the level of health service use and the patient's own financial affordability (36). People with multiple chronic diseases must have richer information and flexibility in order to better manage personal health, otherwise they may be faced with more complicated situation (37). Therefore, in order to clarify the relationship between people with multiple chronic diseases (MCC) and various factors, and to obtain more information that is conducive to MCC self-care decision-making, this study divides chronic disease patients into monopathy and multimorbidity groups. On this basis, the impact of the included variables on the health-related quality of life are investigated when patients suffer from a single chronic disease or multiple chronic diseases. The results are shown in Table 6.

**Table 6:** Influencing factors of HRQOL in PCD in five districts: comparison of monopathy and multimorbidity.

**Variable**	**Monopathy**	**Multimorbidity**
**Drug accessibility**	0.00540^*^	0.0565^***^
**OOP level**	0.0359^***^	0.0748^***^
**Gender** (based on Male)		
Female	−0.0150^**^	−0.0782^***^
**Age**	−0.00447^***^	−0.0100^***^
**BMI** (based on underweight)		
Normal	0.0681^***^	0.111^***^
Overweight	0.0928^***^	0.0254^***^
**Marital status** (based on unmarried/divorced/widowed)		
Married	0.0351^***^	0.140^***^
**Education level** (based on Below junior high school education)		
Junior high school and above	−0.0316^***^	0.101^***^
**Income level** (based on 0–999)		
1000–1999	0.0297^***^	0.155^***^
2000–2999	0.0350^***^	0.158^***^
3000 and more	0.0466^***^	0.304^***^
**Health insurance** (based on Urban residents basic health insurance)		
Urban workers basic health insurance	0.0216^**^	−0.110^***^
New rural cooperative health insurance	0.0364^***^	−0.000873^***^
Others	−0.0258^*^	−0.150^***^
**Health file** (based on Yes)		
No	0.00964	−0.0422^***^
Unknown	−0.0692^***^	−0.0920^***^
**Province** (based on Gansu province)		
Hebei	0.0116	0.193^***^
Sichuan	0.0380^***^	0.301^***^
Tianjin	0.0839^***^	0.0774^***^
Zhejiang	0.0787^***^	0.0560^***^
_cons	0.861^***^	0.677^***^

t statistics in parentheses

* *p < 0.05*,

** *p < 0.01*,

*** *p < 0.001*.

The results show that the health-related quality of life of patients with multimorbidity is more affected by various variables than patients with monopathy. From the perspective of drug-related factors, the higher the patient's accessibility to the drugs needed for the treatment of chronic diseases is, the greater the positive impact on the patient's health utility value is, and the impact on patients with multimorbidity is significantly greater than that on patients with monopathy. From the perspective of the affordability of patient's out-of-pocket expenses, the heavier the OOP economic burden is, the greater the impact on the patient's health utility value is, and the impact on patients with multimorbidity is slightly greater than that on patients with monopathy.

## Discussion

In this sample of patients with chronic diseases in five districts in China, we use the latest EQ-5D-5L to assess their quality of life. Through the CLAD regression model, our study identifies the sociodemographic factors that affect health-related quality of life, and clarifies the significant impact of drug accessibility factors and out-of-pocket burden factors on HRQoL. Through differential analysis, we compare the difference in health effects between patients with single disease and those with multiple diseases.

In recent years, as more and more treatment costs are transferred to patients in various forms, such as high deductibles, co-payments and co-insurance, the issue of “financial toxicity” in healthcare is receiving great attention. The term summarizes the harmful effects of the economic burden of medical expenditures on patients, and also includes the unintended consequences of medical costs: such as the trade-off between essential healthcare, barriers to higher quality of life, and non-health-related necessities (12). These problems are particularly relevant to patients, especially those with poor socioeconomic status, who may adjust their treatment plans due to unaffordable costs. Out-of-pocket medical expenses add to the suffering of people with chronic diseases because they require long-term care and rely on multiple and regular use of services. These sufferings span financial and psychological fields and are ultimately reflected in the patient's health-related quality of life (12). This study finds that the increase in OOP affordability significantly improves the health-related quality of life of patients with chronic diseases, which is consistent with previous findings (25). This is precisely because out-of-pocket expenses, a health-related stressor, usually damage other aspects of patients' lives, such as self-perception of health, mental health, and health-related quality of life (12). Therefore, future research should specifically assess the economic difficulties associated with chronic diseases, and expand educational interventions for clinicians and patients to improve compliance. Paying attention to patients' willingness to include out-of-pocket expenses in treatment decisions and patients' mental health to reduce the financial burden and psychological pressure of patients. China can fully promote product competition by encouraging generic policies and substitution of generic drugs. Price negotiation and volume-based procurement can strengthen medical insurance's control over drug prices and promote price competition. By reducing import tariffs, the distribution price difference can be reduced, ensuring that patients with chronic diseases can purchase drugs at affordable prices.

Due to the fragility of social status, patients with chronic diseases are usually in the most disadvantaged position in terms of access to medicines, which will directly affect their health and medicine welfare effects (38). There are 14 out of every 100 cases in the Manaus population without access to medication, which is more common in people with poor health and chronic diseases (39). Based on this, this study explores the relationship between drug accessibility and HRQoL of patients with chronic diseases, and finds that the increase in the accessibility of medicines has significant positive impact on the health utility value of patients with chronic diseases. This assumption will be in line with other studies: Kuwaiti patients with rheumatoid arthritis have significantly reduced joint swelling and reduced disease activity due to the higher rate of biomedicine use, and the health assessment questionnaire scores higher (40). In order to ensure the accessibility of medicines, a number of studies have provided suggestions for national medicine policies. The study in Vietnam (41) indicated that it was necessary to combine national pricing and antibacterial management policies to ensure the accessibility of antibacterial drugs; A Swiss study (42) found that the time period from approval to inclusion in the “special list” was very important for assessing the accessibility of medicines for cancer patients. It proposed that priority should be given to price negotiations for cancer drugs with high clinical benefits, or the maximum period of negotiation should be set to ensure drug accessibility for patients. China's new “Drug Administration Law” has added the goal of “guaranteeing access to medicines”, which requires that patients should also be guaranteed timely access to relevant information about rational drug use besides supply channels for essential drugs, such as medical institutions and retail pharmacies. In this regard, priority should be given to review and approval of clinically urgently needed drugs in short supply. Implementing a drug reserve system and a supply and demand testing system to ensure the accessibility of drugs under emergency conditions. It is clearly stipulated that pharmacists be responsible for rational medication guidance and strengthening the publicity and education of drug safety to the masses.

In this study, the average quality of life utility score for patients with chronic diseases is 0.727 (range −0.391 to 1), which is slightly lower than the value reported by other studies (43). This may be because most of our respondents are elderly. There are significant differences in the dimensions of pain or discomfort, anxiety, or depression among patients with chronic diseases in five districts. Compared with the other three dimensions, there are more people who have problems in the two dimensions of pain or discomfort and anxiety or depression. This is owing to the long disease process and heavy economic burden, and chronic diseases are difficult to cure and easy to relapse (44). Physical discomfort and mental discomfort caused by illness or drugs can also lead to the situation. On the whole, the health-related quality of life of patients with chronic diseases in Sichuan Province is significantly higher than that of the other four provinces and cities. This may be due to the relatively low living pressure and relatively high air quality in Sichuan Province compared to Tianjin and Zhejiang, and the relatively high level of economic development compared to Hebei and Gansu Provinces.

Old age is negatively correlated with HRQoL, which is consistent with previous studies (45). This may be related to women's physical fitness and physical strength, and their own pain tolerance is weaker than that of men (46, 47). Our research finds that overweight can improve the health utility value of patients with chronic diseases compared to emaciation and being of normal weight. Weakness of the physique of patients with chronic diseases and reduced nutrient absorption capacity should be considered (48). Different from previous studies on education and health-related quality of life (49), in our study, patients with low education have higher health utility values. On the one hand, this may be related to the poor understanding of health standards and low self-health expectations of people with low academic qualifications. On the other hand, the bias brought by the sample should also be considered. However, for patients with multimorbidity, the level of education is positively correlated with health effectiveness, indicating that patients with multiple chronic diseases need more knowledge of health management. There is ample evidence for the relationship between income and health-related quality of life (50, 51). Our research further concludes that: In China, patients with a monthly income of <1,000 RMB would have lower HRQoL due to poor quality of life such as diet and living conditions. At the same time, we should also consider the impact of high medical costs on chronic diseases of low-income people. We find that urban workers health insurance is associated with higher HRQoL. In other studies (52, 53), all the three types of medical insurance are helpful to the health-related quality of life of patients with chronic diseases. In addition, the impact of the three types of insurance is not very different, indicating that the medical insurance policy has a certain degree of fairness for people with chronic diseases. By increasing the detection rate of chronic diseases and realizing early treatment of chronic diseases, HRQoL can be improved (54).

Studies have shown that 42.4% of Chinese elderly patients with chronic diseases suffer from multiple chronic diseases (55). Although patients with chronic diseases only account for a small part of the sample in this study, multimorbidity is still a complex situation faced by many chronic disease patients. Studies have shown that patients with more comorbidities have better diagnosis of chronic diseases, but it does not improve the control of these diseases (56). Patients with multiple chronic diseases are high users of health services and have an increased risk of adverse health outcomes. Therefore, improving access to medical services for patients with multiple diseases is a priority of the healthcare system (56). This study explores the differential factors affecting health-related quality of life of patients with single disease and multiple diseases through heterogeneity analysis, and finds that the health-related quality of life of patients with multiple chronic diseases is more affected by various variables than those with single disease. It shows that patients with multiple diseases are more sensitive and vulnerable. Based on the heavy burden of multiple diseases and the high demand for health services, China's future health system development should shift from preventing and controlling chronic diseases as individual diseases to meeting the comprehensive health needs of people with multiple diseases (57).

Our research has some innovative and policy implications. First of all, compared with previous studies using EQ-5D-5L (16, 58), we use this questionnaire for the first time to evaluate the health-related quality of life of multiple chronic disease groups in China, and apply the CLAD model to avoid the ceiling effect. Compared with previous health surveys (SF-36) (29, 59), EQ-5D-5L proved to be more friendly to less educated respondents, especially in rural China (30). As the largest developing country, the current status of health-related quality of life of patients with chronic diseases and its influencing factors in China are worthy of attention for future pharmaceutical policies and health poverty alleviation policies. Secondly, the value set based on other countries (such as Japan or the United Kingdom) (60) may be biased when applied to the health-related quality of life measurement of patients with chronic diseases in China on account of cultural differences. We have adopted the newly developed Chinese version value set, which has good population applicability. Thirdly, for the first time, the out-of-pocket burden of patients with chronic diseases, and the drug accessibility are included in the scope of health-related quality of life, prompting special attention to accessibility and affordability of medicines for patients with chronic diseases.

Our research has some limitations. First, our sample size is limited, and the respondents' regional choices are limited. All interviewees are from five districts in China. Therefore, the results of this study may not be representative of all patients with chronic diseases in China. However, we select districts that can represent the east, middle, and west of China based on the economic level and the allocation of health resources, in order to reduce the bias in the research results caused by regional restrictions. Therefore, this study can be used as a reference for similar regions or wider regions. In addition, this is a cross-sectional study that can only introduce factors that affect the HRQoL of patients with chronic diseases, but cannot provide evidence of causality. In addition, the study does not include HRQoL studies and comparative studies for patients with specific chronic diseases.

## Conclusion

Through the analysis of the research results, we conclude that the CLAD model is better than the Tobit model for the regression results of the health-related quality of life of patients with chronic diseases. Reducing out-of-pocket expenses of patients with chronic diseases, especially in poor areas and in patients with relatively poor socioeconomic status, is a positive factor in improving the quality of life of patients with chronic diseases. At the same time, we believe that strengthening the protection of public health services and the supply of medicines and increasing the basic welfare of patients with chronic diseases are also very important for promoting and improving the mental health of patients with chronic diseases. At the same time, future focus should shift from preventing and controlling chronic diseases as individual diseases to meeting the comprehensive health needs of people suffering from multiple diseases.

## Data Availability Statement

The raw data supporting the conclusions of this article will be made available by the authors, without undue reservation.

## Ethics Statement

The studies involving human participants were reviewed and approved by Ethics Committee of Nanjing Hospital of Chinese Medicine affiliated with Nanjing University of Chinese Medicine. Written informed consent to participate in this study was provided by the participants' legal guardian/next of kin.

## Author Contributions

Study conception and design were performed by ST. Data analysis was performed by ML and DY. The first draft of the manuscript was written by YG. KT checked the data and edited the language. All authors commented on previous versions of the manuscript, contributed to the material preparation and data collection, and read and approved the final manuscript.

## Conflict of Interest

The authors declare that the research was conducted in the absence of any commercial or financial relationships that could be construed as a potential conflict of interest.
