# Supplementary material for: Do Drug Accessibility and OOP Burden Affect Health-Related Quality of Life of Patients With Chronic Diseases? — EQ-5D-5L Evaluation Evidence From Five Districts in China
Source: Front Public Health. 2021 Mar 12;9:656104. doi: 10.3389/fpubh.2021.656104 (PMC8006263; doi:10.3389/fpubh.2021.656104)
Supplement: Supplementary file 1 [file Data_Sheet_1.docx]

**Appendix1 Basic characteristics of PCD in five districts**

| tloVariable | | Gansu | | Hebei | | Sichuan | | Zhejiang | | Tianjin | | Total | |
| --- | --- | --- | --- | --- | --- | --- | --- | --- | --- | --- | --- | --- | --- |
|  |  | Number | Percentage(%) | Number | Percentage(%) | Number | Percentage(%) | Number | Percentage(%) | Number | Percentage(%) | Number | Percentage(%) |
| **Age** | 0-49 | 64 | 19.7 | 34 | 16.8 | 25 | 13.0 | 31 | 14.7 | 58 | 46.8 | 212 | 20.1 |
|  | 50-59 | 74 | 22.8 | 53 | 26.2 | 28 | 14.5 | 24 | 11.4 | 13 | 10.5 | 192 | 18.2 |
|  | 60-69 | 84 | 25.8 | 56 | 27.7 | 69 | 35.8 | 64 | 30.3 | 22 | 17.7 | 295 | 28.0 |
|  | 70 or more | 103 | 31.7 | 59 | 29.2 | 71 | 36.8 | 92 | 43.6 | 31 | 25.0 | 356 | 33.7 |
| **Gender** | Male | 148 | 45.5 | 99 | 49.0 | 73 | 37.8 | 105 | 45.6 | 56 | 45.2 | 481 | 45.6 |
|  | Female | 177 | 54.5 | 103 | 51.0 | 120 | 62.2 | 106 | 54.4 | 68 | 54.2 | 574 | 54.4 |
| **BMI**  **Category** | Wasting | 41 | 12.6 | 18 | 8.9 | 20 | 10.4 | 30 | 14.2 | 38 | 30.6 | 147 | 13.9 |
|  | Normal | 175 | 53.8 | 95 | 47.0 | 107 | 55.4 | 115 | 54.5 | 44 | 35.5 | 536 | 50.8 |
|  | Overweight | 109 | 33.5 | 89 | 44.1 | 66 | 34.2 | 66 | 31.3 | 42 | 33.9 | 372 | 35.3 |
| **Marital Status** | Married | 282 | 86.8 | 180 | 89.1 | 161 | 83.4 | 179 | 84.8 | 64 | 51.6 | 866 | 82.1 |
|  | Unmarried  /divorced  /widowed | 43 | 13.2 | 22 | 10.9 | 32 | 16.6 | 32 | 15.2 | 60 | 48.4 | 189 | 17.9 |
| **Education Level** | Below junior high school education | 226 | 69.5 | 131 | 64.9 | 152 | 78.8 | 152 | 72.0 | 74 | 59.7 | 735 | 69.7 |
|  | Junior high school education and above | 99 | 30.5 | 71 | 35.1 | 41 | 21.2 | 59 | 28.0 | 50 | 40.3 | 320 | 30.3 |
| **Income Level** | 0-999 | 130 | 40.0 | 78 | 38.6 | 54 | 28.0 | 38 | 18.0 | 55 | 44.4 | 355 | 33.6 |
|  | 1000-1999 | 37 | 11.4 | 25 | 12.4 | 53 | 27.5 | 32 | 15.2 | 8 | 6.5 | 155 | 14.7 |
|  | 2000-2999 | 93 | 28.6 | 45 | 22.3 | 39 | 20.2 | 38 | 18.0 | 30 | 24.2 | 245 | 23.2 |
|  | 3000 and more | 65 | 20.0 | 54 | 26.7 | 47 | 24.4 | 103 | 48.8 | 31 | 25.0 | 300 | 28.4 |
| **Health insurance Categories** | Urban residents basic health insurance2 | 107 | 32.9 | 82 | 40.6 | 74 | 38.3 | 81 | 38.4 | 47 | 37.9 | 391 | 37.1 |
|  | Urban workers basic health insurance1 | 41 | 12.6 | 26 | 12.9 | 57 | 29.5 | 50 | 23.7 | 47 | 37.9 | 221 | 20.9 |
|  | New rural cooperative health insurance3 | 164 | 50.5 | 87 | 43.1 | 56 | 29.0 | 61 | 28.9 | 13 | 10.5 | 381 | 36.1 |
|  | Others | 13 | 4.0 | 7 | 3.5 | 6 | 3.1 | 19 | 9.0 | 17 | 13.7 | 62 | 5.9 |
| **Whether there is a health file** | Yes | 55 | 16.9 | 32 | 15.8 | 50 | 25.9 | 87 | 41.2 | 28 | 22.6 | 252 | 23.9 |
|  | No | 232 | 71.4 | 115 | 56.9 | 78 | 40.4 | 57 | 27.0 | 45 | 36.3 | 527 | 50 |
|  | Unknown | 38 | 11.7 | 55 | 27.2 | 65 | 33.7 | 67 | 31.8 | 51 | 41.1 | 276 | 26.2 |
